# Single-trial classification of motor imagery differing in task complexity: a functional near-infrared spectroscopy study

**DOI:** 10.1186/1743-0003-8-34

**Published:** 2011-06-18

**Authors:** Lisa Holper, Martin Wolf

**Affiliations:** 1 Biomedical Optics Research Laboratory (BORL), Division of Neonatology, Department of Obstetrics and Gynecology, University Hospital Zurich, Frauenklinikstrasse 10, 8091 Zurich, Switzerland; 2 Institute of Neuroinformatics (INI), University of Zurich and ETH Zurich, Winterthurerstrasse 190, 8057 Zurich, Switzerland

**Keywords:** wireless functional near-infrared spectroscopy (fNIRS), motor imagery, motor execution, single-trial classification, linear discriminant analysis, brain computer interface (BCI)

## Abstract

**Background:**

For brain computer interfaces (BCIs), which may be valuable in neurorehabilitation, brain signals derived from mental activation can be monitored by non-invasive methods, such as functional near-infrared spectroscopy (fNIRS). Single-trial classification is important for this purpose and this was the aim of the presented study. In particular, we aimed to investigate a combined approach: 1) offline single-trial classification of brain signals derived from a novel wireless fNIRS instrument; 2) to use motor imagery (MI) as mental task thereby discriminating between MI signals in response to different tasks complexities, i.e. simple and complex MI tasks.

**Methods:**

12 subjects were asked to imagine either a simple finger-tapping task using their right thumb or a complex sequential finger-tapping task using all fingers of their right hand. fNIRS was recorded over secondary motor areas of the contralateral hemisphere. Using Fisher's linear discriminant analysis (FLDA) and cross validation, we selected for each subject a best-performing feature combination consisting of 1) one out of three channel, 2) an analysis time interval ranging from 5-15 s after stimulation onset and 3) up to four Δ[O_2_Hb] signal features (Δ[O_2_Hb] mean signal amplitudes, variance, skewness and kurtosis).

**Results:**

The results of our single-trial classification showed that using the simple combination set of channels, time intervals and up to four Δ[O_2_Hb] signal features comprising Δ[O_2_Hb] mean signal amplitudes, variance, skewness and kurtosis, it was possible to discriminate single-trials of MI tasks differing in complexity, i.e. simple versus complex tasks (inter-task paired t-test p ≤ 0.001), over secondary motor areas with an average classification accuracy of 81%.

**Conclusions:**

Although the classification accuracies look promising they are nevertheless subject of considerable subject-to-subject variability. In the discussion we address each of these aspects, their limitations for future approaches in single-trial classification and their relevance for neurorehabilitation.

## 1 Introduction

Direct neural interfaces, i.e. brain computer interfaces (BCIs), can provide users in neurorehabilitation, such as individuals with severe brain disorders, with basic communication capabilities or the control over external devices through their mental processes alone, bypassing the muscular system [1]. To develop a given method for use in BCI systems, a reliable single-trial classification of the brain signals derived from mental activation is important for this purpose and this was the aim of the presented study.

A relatively new method that has only recently attracted researchers' attention in the context of neural interface development is functional near-infrared spectroscopy (fNIRS). fNIRS is a non-invasive technique based on neurovascular coupling, which uses the tight coupling between neuronal activity and localized cerebral blood flow to monitor hemodynamic changes associated with cortical activation [2]. Hence, in contrast to traditional neural interfaces approaches based on electroencephalography (EEG) that rely on electrical brain signals, fNIRS relies on the measurement of the task-induced hemodynamic changes in the cortex, similar to those signal obtain in functional magnetic resonance imaging (fMRI). This study presents an attempt of offline classification of single trials derived from a novel developed wireless fNIRS instrument [3].

### 1.1 Single-trial classification of fNIRS data

Previous studies investigating single-trial classifications of fNIRS hemodynamic data included different combinations of mental tasks, signal features and classifiers. Sitaram et al. [4] performed offline classification of hand motor imagery (MI) using mean amplitude changes in Δ[O_2_Hb] and Δ[HHb] as the class discriminatory features; a maximum accuracy of 89% was achieved using a hidden Markov model (HMM). Coyle et al. [5] performed online classification by asking subjects to control a binary switch by modulating changes in mean Δ[O_2_Hb] over the motor cortex and achieved 50-85% accuracy in online trials. Naito et al. [6] investigated over the prefrontal cortex in locked-in patients who were requested to perform different high-level mental tasks corresponding to 'yes' and 'no' in response to a series of questions. An average offline classification accuracy of 80% was achieved in 40% of the locked-in participants using maximum and mean Δ[O_2_Hb] as features and a non-linear discriminant classifier. Tai and Chau [7] classified offline visually-cued positively and negatively emotional induction tasks. Using mean Δ[O_2_Hb] amplitude, variance, skewness and kurtosis as features combined with linear discriminant analysis (LDA) and support vector machine (SVM) classifiers the authors achieved accuracies upwards of 75.0%. Luu and Chau [8] decoded neural correlates of decision making by asking subjects to mentally evaluate two possible drinks and decide which they preferred. Using mean Δ[O_2_Hb] amplitude as feature and Fisher's linear discriminant analysis (FLDA), they achieved an average accuracy of 80%.

### 1.2 Motor imagery as mental task

In this study we aimed to focus on the offline classification of single trials derived from kinaesthetic MI. MI is described as the mental rehearsal of voluntary movement [9]. According to the so-called simulation hypothesis [10,11], MI activates a cortical network located in primary motor cortex (M1) and secondary motor areas, such as premotor cortex (PMC), supplementary motor area (SMA) and parietal cortices [12] which is thought to overlap with those areas responsible for motor execution (ME) of the same motor action [13,14]. Besides its relevance in BCI development, decoding MI signals is particularly appealing from a neurorehabilitation perspective. Due to its effect on brain activation MI is thought to access the motor network independently of motor recovery even in patients with impaired or paralysed motor function. MI could therefore be integrated into usual neurorehabilitative training [15] with or without combination with neural interface applications [16,17].

Further, to use a certain MI task for such purposes, it is of major advantage if the given method not only detects related signal changes, but also that it differentiates between different degrees of complexity of a given task. In addition, for future BCI applications the potential signal parameters of those tasks that allow for differentiation between simple versus complex tasks are then required to be classified on the single-trial level. In this study, we therefore aimed to extend previous studies by addressing this combined approach in evaluating the classification of two MI tasks differing in complexity, i.e. simple and complex finger-tapping tasks; these tasks closely correspond to tasks used in various fMRI studies and those investigating patients in neurorehabilitation [18-21]. To test this we made use of a novel wireless fNIRS instrument that we have previously tested to be capable of detecting oxygenation changes in response to MI [22,23].

Taken together, in the presented study, we aimed to investigate a combined approach which has not been addressed in this extent by previous studies using fNIRS: 1) offline single-trial classification of brain signals derived from a novel wireless fNIRS instrument using a simple combination of features and Fisher's linear discriminant analysis (FLDA) as classifier aimed to 2) discriminate between MI signals in response to different tasks complexities, i.e. simple and complex MI tasks. This paper aims to describe our findings and to discuss the potential relevance and limitations of our observations for future neurorehabilitative applications.

## 2 Materials and methods

### 2.1 Subjects

12 healthy subjects were included (6 males, mean age 29 years, range 26 - 33 years). Exclusion criteria were any history of visual, neurological or psychiatric disorders or any current medication. All subjects gave informed consent. All subjects had normal or corrected-to-normal vision. The study was approved by the ethics committee of the Canton of Zurich and was in accordance with the latest version of the Helsinki declaration.

All subjects were right-handed (mean Laterality Quotient (LQ) of 83, range 72 - 100; mean deciles level of 6.6, range 4 - 10) according to the Edinburgh Handedness Inventory (EHI) [24]. The self-administered Vividness of Movement Imagery Questionnaire (VMIQ) [25] revealed an overall relative imagery ability of 82.43 ± 13.21 (range 73 - 107). Compared with the cut-off-point established by Whetstone [26] that estimates imagery ability in relation to a total score of 75, eight of our subjects had a comparatively good and four subjects a lower imagery ability.

### 2.2 Experimental protocol

Each subject participated in one session. All experiments were conducted in a quiet room. Subjects were asked to sit in front of a LCD monitor (94 cm diagonal, 1366 × 768 pixels) at a comfortable distance of approximately 60 cm from the eyes. A wireless numerical keyboard (Logitech^® ^Cordless Number Pad) was placed in front the subjects.

#### 2.2.1 Motor imagery (MI) tasks

The experiment consisted of the following two task conditions:

• MI-simple: subjects were asked to imagine a simple finger-tapping task by repetitively pressing button 'zero' (0) of a number keyboard using their thumb of the right hand with a frequency of approximately 3 Hz. The start of the trial was indicated by a visual stimulus 'GO - 0' on the screen.

• MI-complex: subjects were asked to imagine a complex sequential finger-tapping task by repetitively pressing a predefined sequence on the keyboard using all fingers of their right hand with the same frequency as in MI-simple. The sequence was presented at the start of the trial on the screen: e.g. 'GO - 2-2-5-3-4'. The number stimuli symbolized the numbered fingers of a hand, 1 = thumb, 2 = index finger, 3 = middle finger, 4 = ring finger and 5 = little finger. For example, the sequence 2-2-5-3-4 indicated the following task: index finger twice, little finger once, middle finger once, and ring finger once. Five sequences of similar complexity were presented in a randomized order each comprising five tapping acts. This task is similar to that used in various fMRI studies of stroke and stroke recovery [18-21].

**Figure 1:**
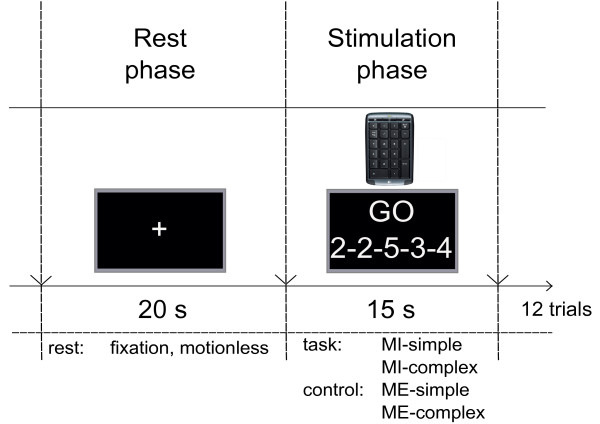
**Experimental design**. An example of the trial layout showing the stimulation periods (15 s) alternating with the rest periods (20 s) during which subjects had to either execute or imagine finger tapping on a keyboard. Start of the stimulation was indicated by the word GO.

Prior to recording, subjects completed a practice trial to familiarise with and properly understand the tasks. An example of the trial layout is shown in Figure 1: in total, 12 trials of each condition consisting of stimulation phases (15 s) were presented alternating with rest phases (20 s); resulting in 24 trials per subject with a total duration of 14 min. During the rest phases a fixation cross was presented and subjects were instructed to simply watch the screen and remain motionless. All trials were randomized between the two tasks and between the five different task sequences. Subjects were reminded to perform the executed and imagined movements as precise and as fast as possible. All finger-tapping tasks were self-paced, however subjects were asked to perform finger-tapping with frequencies of approximately 2 Hz. Stimuli were presented using white numbers on the screen generated by the software Presentation^® ^(Neurobehavioral systems, Albany, USA).

Subjects were asked to use kinesthetic MI (i.e. individuals using imagery to imagine how movements feel, supposedly associated with kinesthetic feeling) since recent studies demonstrated that kinesthetic rather than visual imagery (i.e. individuals imagine watching themselves performing a task) modulates cortico-motor excitability [27,28].

#### 2.2.2 Control motor execution (ME) measurements

After the experiment, subjects were asked to complete two additional motor control measurements 1) to verify the right positioning of the fNIRS instrument (see details of positioning in the next section 2.3) and 2) to support our hypothesis that the complex task was indeed more difficult than the simple task. The control ME measurements were conducted after the MI tasks to avoid potential performance interference with a previous execution of the imagined movements. They consisted of the same conditions applied in the MI tasks (Figure 1).

• ME-simple: same as MI-simple, but subjects were asked to actually perform the simple task by pressing button 'zero' (0) on the keyboard repetitively using their thumb over the whole stimulation phase with a frequency of approximately 3 Hz.

• ME-complex: same as MI-simple, but subjects were asked to actually perform the complex task by pressing five buttons on the keyboard using all fingers in the same predefined sequences and frequency as presented in MI-complex.

Timing and procedures were identical to the MI conditions. All tasks were carried out using the wireless numerical keyboard (Logitech^® ^Cordless Number Pad) which allowed recording of all keystrokes of all five fingers; data were transferred to PC via USB and stored for further analysis.

### 2.3 fNIRS measurements

fNIRS is a non-invasive technique based on neurovascular coupling, which exploits the effect of metabolic activity due to neural processing on the oxygenation of cerebral tissue. Utilizing this tight coupling between neuronal activity and localized cerebral blood flow, fNIRS measures hemodynamic changes associated with cortical activation, i.e. typically an increase in oxy-hemoglobin concentration Δ[O_2_Hb] and a decrease in deoxy-hemoglobin concentration Δ[HHb] [2]. The Δ[O_2_Hb] change usually has considerably higher amplitude than the Δ[HHb] change and also a higher contrast to noise ratio. The reason is that while an increased O_2_-consumption reduces Δ[O_2_Hb], both the concurrent increased cerebral blood flow and volume lead to an increase in Δ[O_2_Hb]. For Δ[HHb] the increase in blood flow and volume lead to opposite effects and thus, the total change in Δ[HHb] has a smaller amplitude [29].

**Figure 2:**
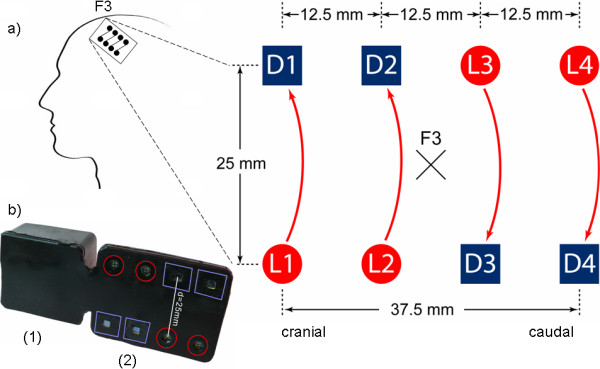
**Wireless fNIRS sensor**. a) Top-view: schematic of light sources (L1, L2, L3, and L4) and detectors (D1, D2, D3, and D4) on the sensor. b) Wireless fNIRS sensor with casing; (red) light sources, (blue) detectors, (1) analog and wireless communications and power-supply electronics, (2) optical probe [3]. The centre of the sensor was positioned presumably covering position F3 according to the 10-20 system [30]. Three channels were considered for analysis. D1-L1 was positioned in cranial direction, D4-L4 in caudal direction.

fNIRS was recorded using a novel miniaturized fNIRS sensor previously described in detail [3]. This wireless and portable fNIRS sensor does not require the subject's body or head to be restrained, and therefore can be used as a brain monitoring tool in everyday environments. The sensor components are mounted onto a four-layer rigid-flexible printed circuit board (PCB) which, in combination with a highly flexible casing made of medical grade silicone, enables the sensor to be aligned to curved body surfaces such as the head. The size of the device is 92 × 40 × 22 mm and weighs 40 g. The optical system comprises four light sources at two different wavelengths (760 nm and 870 nm) and four detectors (PIN silicon photodiodes) with a source-detector distance of 12.5 mm (Figure 2). The power is provided by a rechargeable battery, which allows a continuous data acquisition for 180 minutes at full light emission power. The light intensity is sampled at 100 Hz and the resulting data are transmitted wirelessly to the host computer by Bluetooth. The operating range of the sensor is about 5 m.

For fNIRS recording, one sensor was placed over the subject's left hemisphere over F3 according to the international 10-20 system [30]. With the compact sensor of 37.5 mm length and 25 mm width, we assumed to cover secondary motor areas, presumably including PMC and SMA. Cortical activation in these areas has been previously described during MI performance [31,32]. The sensor was fixed on the subject's head using self-adhesive bandages (Derma Plast CoFix 40 mm, IVF Hartmann, Neuhausen, Switzerland).

### 2.4 EMG measurements

Surface electromyogram (EMG) was monitored bilaterally in combination with fNIRS in all subjects to confirm the absence of muscle activity during the MI tasks. EMG was obtained using a customisable asymmetrical dual channel digital EMG unit (NeuroTrac™ ETS, Verity Medical Ltd., Romsey, Hampshire, United Kingdom) that detects electrical activity from 0.2 μV up to 2000 μV. One pair of electrodes was placed over musculus extensor digitorum muscles to measure (1) the activity during the MI tasks, (2) the level of muscle activity during the rest phases and (3) the timing and frequency of the finger-tapping during the ME control measurements. After each session, EMG data were graphically displayed and visually reviewed for task-unrelated movements using the automated EMG software application (Verity Medical Ltd., NeuroTrac™EMG Software). In all recorded subjects, EMG graphics showed that subjects performed the right hand button presses during the ME control measurements with a suitable timing and frequency; activity was lower during the rest phase compared to the active stimulation phases; there was no activity recorded in the left (unused) hand during both ME controls (< 20 μV). During the MI tasks, EMG of both forearms showed a constant electrical activity below < 20 μV. In two subjects the electrical activity of the right forearm seemed to be higher and more variable in the MI-complex task than in MI-simple, but still < 20 μV.

## 3 Data analysis

### 3.1 Data pre-processing

By measuring intensity of NIR light after its transmission through tissue, it is possible to determine oxygenation changes over time of oxy-hemoglobin (O_2_Hb) and deoxy-hemoglobin (HHb), which represent the dominant light absorbers for living tissue in the NIR spectral band. By applying the modified Beer-Lambert law (MBLL), the concentration for O_2_Hb and HHb ([O_2_Hb], [HHb]) were computed from the measured absorption changes [33,34].

A program for MATLAB^® ^(Version 2008a) was written and applied to pre-process the raw light intensity values and to compute [O_2_Hb] and [HHb] changes. The measurement files that were acquired during the fNIRS experiment containing the intensity signals of the NIR light, sampled at 100 Hz for all combinations of light-sources, wavelengths and detectors, as well as the intensity of the ambient light. The program subtracts the ambient light intensities from the fNIRS measurement values before low-pass filtering (7th order Chebyshew with 20 dB attenuation at 5 Hz) and decimates the signals to a sampling rate of 10 Hz. Consecutively, the MBLL is used to compute the changes of [O_2_Hb] and [HHb] applying differential path lengths factors (DPF) of 6.75 for the 760 nm and 6.50 for the 870 nm light-sources [35]. The linear signal drift is then subtracted from the resulting [O_2_Hb] and [HHb] signals.

Source-detector combinations (channels) that did not show significant oxygenation changes in individual subjects were excluded from further analysis, since it was assumed that those channels did not cover the activated cerebral region at all. For this reason the fourth channel was excluded from analysis as its more lateral location was prone to high artifacts and had a very low signal to noise ratio. Further, subjects that did not show significant oxygenation changes (p > 0.05) in all channels in the ME control measurements and the MI tasks were excluded from analysis.

Consecutively, dependent variables for further statistical analysis were derived from the non-excluded [O_2_Hb] and [HHb] datasets. Specifically, the mean of the stimulation phases ([HHb]_stim_, [O_2_Hb]_stim_,) and the mean of the rest phases ([HHb]_rest_, [O_2_Hb]_rest_, baselines) were considered, calculated for each trial and channel per subject. The statistical significance of the intra-condition differences between ([HHb]_rest_, [O_2_Hb]_rest_) and ([HHb]_stim_, [O_2_Hb]_stim_), later referred to as Δ[HHb] and Δ[O_2_Hb], was analyzed over channels 1-3 for each condition, each subject in the control ME tasks and the MI conditions using the paired t-test (CI 95%, alpha level p ≤ 0.005, power p = 0.764). The signal-to-noise ratio (SNR, defined as the ratio of the mean signal to its standard deviation) was calculated to evaluate the signal strength within each channel.

### 3.2 Single-trial classification of MI signals

Single-trial classification was performed of the hemodynamic signals obtained after processing using SPSS (Version 16.0). Previous studies have either classified light intensity directly [6] or converted the signals to haemoglobin concentrations [4] prior to classification. Since it has not been shown that one method is more discriminating than the other, we classified the processed optical signals.

The goal of the classification was to discriminate the two MI tasks based on single-trial signals. In particular, we aimed to classify Δ[O_2_Hb] signals derived from the difference between the baselines phases (20 s) and the stimulation phases (15 s) of each single-trial into one of the two tasks (MI-simple or MI-complex). The classification was based on the definition of a best-performing combination for each subject consisting of: 1) a specific channel, 2) a specific analysis time interval within the stimulation phase and 3) a set of up to four signal features.

1. Channels: each of the channels 1-3 were tested separately for each subject and the best-performing channel was selected.

2. Analysis time intervals: each time interval within the stimulation phase (0-15 s in Figure 1) was defined by a start time and an end time. Start times ranged from 1 - 11 s in 1 s increments, while end times spanned from 5 - 15 s, also in 1 s increments. All possible combinations of start and end times were considered as valid time intervals for classification. These start and end times were considered according to the typical time course of the hemodynamic response delay after stimulation onset [36,37].

3. Features: the following four features were selected from those previously published and tested by [7]. All features were calculated for each subject (N = 12 subjects) and each trial (N = 12 trials):

○ Mean: average signal amplitude.

○ Variance: measure of signal spread.

○ Skewness: measure of the asymmetry of signal values around its mean relative to a normal distribution.

○ Kurtosis: measure of the degree of peakedness of a distribution of signal values relative to a normal distribution.

Using Fisher's linear discriminant analysis (FLDA) all possible classification combinations were tested for each subject. Classification accuracy was evaluated using cross validation. Due to the relatively small size of the feature space, an exhaustive search was performed for each subject, and the best-performing combination was reported.

Two-tailed Pearson's correlation coefficients (*r*) with p-value (significance level *p *≤ 0.05) were calculated to evaluate correlations between the mean values of the four features and the classification accuracy within the selected subjects.

## 4 Results

### 4.1 Control ME measurements

We first analysed the control ME measurements to confirm our assumption that we were indeed recording from motor-related cortical areas, i.e. presumably secondary motor areas relevant for MI performance. Two subjects were excluded at this stage as their data did not show significant Δ[O_2_Hb] increases. In all remaining subjects (N = 12), the control ME measurements elicited significant intra-control differences between baselines and stimulation phases. On the overall-subject-level significant larger averaged amplitudes were observed during ME-complex (Δ[O_2_Hb] 0.453 ± 0.098 μmol/l; Δ[HHb] -0.0675 ± 0.021 μmol/l) as compared to ME-simple (Δ[O_2_Hb] 0.189 ± 0.055 μmol/l; Δ[HHb] -0.032 ± 0.078 μmol/l) (inter-task paired t-test overall channels: Δ[O_2_Hb] *p *= 0.001, Δ[HHb] *p *= 0.012).

The keystroke data were used to confirm our hypothesis that the complex task was indeed more difficult than the simple task. The errors of the individual button presses were defined as any finger taps occurring outside the one of the prescribed sequences and the error rate was defined as the (total number of errors)/(total number of finger taps). Results revealed a lower number of total taps and a larger error rate in ME-complex (mean total taps 706 ± 254, mean error rate 0.09 ± 0.03) as compared to MI-simple (mean total taps 912 ± 165, mean error rate < 0.001) (*p *= 0.023). This finding confirmed our hypothesis and we assumed that if performance of ME-complex was proven as overall more difficult than ME-simple, the same could be expected for the mental effort required in the corresponding MI tasks. Based on this estimated discrimination between simple and complex imagined movements, we expected a facilitation of the following classification.

### 4.2 MI tasks

**Figure 3:**
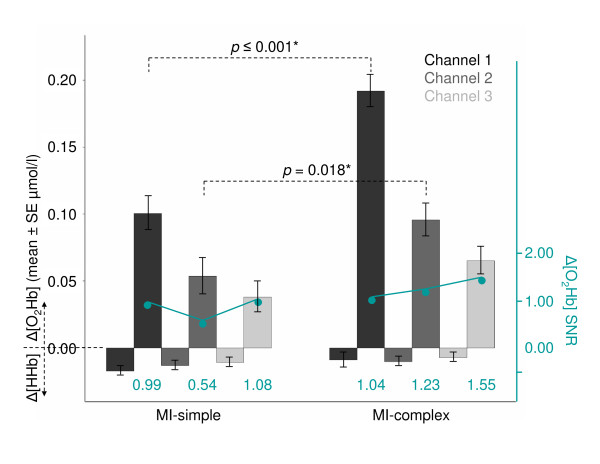
**Mean Δ[O_2_Hb] and Δ[HHb] profile**. Mean Δ[O_2_Hb] and Δ[HHb] (mean ± SE μmol/l) on the overall-subject-level averaged over 12 trials for each channel separately (channel 1 [black], channel 2 [dark gray], channel 3 [light gray]), of the contralateral (right) hemispheres during performance of MI-simple and MI-complex. Shown are also relevant significances of paired t-test (CI 95%, p-values) of Δ[O_2_Hb] between the two tasks. The second y-axis (green) represents the Δ[O_2_Hb] signal-to-noise ratio (SNR, defined as the ratio of the mean signal to its standard deviation) for each channel; the values of each SNR are shown below.

On the overall-subject-level, we first plotted the oxygenation patterns of Δ[O_2_Hb] and Δ[HHb] averaged over all subjects and all trials for each of the channels 1-3. As observed in the control measurements, the same characteristic patterns was found between the two MI tasks reflecting the effect of task complexity (Figure 3, Table 1, **top**): MI-complex (Δ[O_2_Hb] 0.118 ± 0.011 μmol/l; Δ[HHb] -0.009 ± 0.003 μmol/l) revealed larger oxygenation responses as compared to MI-simple (Δ[O_2_Hb] 0.064 ± 0.012 μmol/l; Δ[HHb] -0.014 ± 0.003 μmol/l) (inter-task paired t-test overall channels: Δ[O_2_Hb] *p *= 0.001, Δ[HHb] *p *= 0.029). This was consistent over all channels reaching significance in channel 1 (Δ[O_2_Hb] *p *≤ 0.001) and 2 (Δ[O_2_Hb] *p *= 0.018). In both conditions, channel 1 revealed the largest Δ[O_2_Hb] changes, followed by channel 2 and 3. We suggested that this distribution might be an indicator for the underlying topography, i.e. the cortical regions activated within secondary motor areas: stronger oxygenation changes in the medial (channel 1 and 2) as compared to the more lateral parts (channel 3).

**Table 1:** Mean Δ[O_2_Hb] and Δ[HHb] profiles.

Mean Δ[O_2_Hb] Δ[HHb] Overall-subjects	Channel 1	Channel 2	Channel 3	Overall channels
**MI-simple**				
Δ[O_2_Hb] μmol/l	0.101 ± 0.013	0.054 ± 0.014	0.038 ± 0.011	0.064 ± 0.012
Δ[HHb] μmol/l	-0.017 ± 0.002	-0.0130 ± 0.003	-0.011 ± 0.003	-0.014 ± 0.003
Δ[O_2_Hb] SNR	0.99	0.54	1.08	0.87

**MI-complex**				
Δ[O_2_Hb] μmol/l	0.192 ± 0.012	0.095 ± 0.012	0.065 ± 0.010	0.118 ± 0.011
Δ[HHb] μmol/l	-0.008 ± 0.006	-0.010 ± 0.003	-0.007 ± 0.003	-0.009 ± 0.003
Δ[O_2_Hb] SNR	1.04	1.23	1.55	1.27

**Inter-task paired t-test [simple vs complex]**	**Channel 1**	**Channel 2**	**Channel 3**	**Overall channels**

	Δ[O_2_Hb] (p-values)	Δ[O_2_Hb] (p-values)	Δ[O_2_Hb] (p-values)	Δ[O_2_Hb] (p-values)

**Overall-subjects**	≤ 0.001*	0.018*	0.064	≤ 0.001*

**Subject 1**	≤ 0.001*	≤ 0.001*	≤ 0.001*	≤ 0.001*

**Subject 2**	0.341	≤ 0.001*	≤ 0.001*	≤ 0.001*

**Subject 3**	1.000	0.003*	≤ 0.001*	0.032*

**Subject 4**	1.000	≤ 0.001*	≤ 0.001*	≤ 0.001*

**Subject 5**	≤ 0.001*	≤ 0.001*	≤ 0.001*	≤ 0.001*

**Subject 6**	0.105	0.007*	0.002*	0.046*

**Subject 7**	≤ 0.001*	0.023*	≤ 0.001*	≤ 0.001*

**Subject 8**	0.086	≤ 0.001*	0.004*	0.002*

**Subject 9**	≤ 0.001*	≤ 0.001*	≤ 0.001*	≤ 0.001*

**Subject 10**	0.976	≤ 0.001*	≤ 0.001*	≤ 0.001*

**Subject 11**	0.181	≤ 0.001*	≤ 0.001*	≤ 0.001*

**Subject 12**	0.324	0.026*	≤ 0.001*	0.039*

(Top) Mean Δ[O_2_Hb] and Δ[HHb] (mean ± SE μmol/l) and Δ[O_2_Hb] signal-to-noise ratio (SNR, defined as the ratio of the mean signal to its standard deviation) on the overall-subject-level averaged over channels 1-3 and for each channel separately, of the contralateral (right) hemispheres during performance of MI-simple and MI-complex (Bottom) Inter-task paired t-test (CI 95%, p-values) for each subject of Δ[O_2_Hb] between the two tasks, MI-simple and ME-complex.

Significant values are highlighted with (*).

On the single-subject-level, similar patterns were observed within each subject: all subjects showed a significant effect of task complexity with larger Δ[O_2_Hb] changes in MI-complex as compared to MI-simple (measured overall channels, while in some subjects single channels did not show significant changes, see Table 1, **bottom**); and, in nine subjects (75%) larger Δ[O_2_Hb] changes were found in channel 1 as compared to 2 and 3. Taken together, these findings showed that the individual data contained significant task-related Δ[O_2_Hb] changes within each task and that the simple and complex task could be discriminated.

### 4.3 Classification of MI signals

Using FLDA we classified the MI signals by selecting the best-performing combination based on one channel, a certain time interval and up to four of the features (Δ[O_2_Hb] mean amplitude, variance skewness, kurtosis) for each subject. We concentrated on the Δ[O_2_Hb] signal only, since classification of Δ[HHb] signals did not reveal comparable accuracies. The accuracy of the classification averaged on the overall-subject-level was 81.3 ± 7.0% (range 70.8% - 91.7%) (Table 2). However, considerably subject-to-subject variability was observed in the classification combinations as documented by the following results:

Most frequently selected was channel 3 which might indicate that the data derived from the more medial positioned part of the sensor (channel 1 and 2) were less suitable for discrimination the MI signals investigated in this study. From the analysis on the overall-subject-level we knew that channel 3 elicited smaller overall oxygenation changes as compared to channel 1 and 2. To test why the signal amplitudes in the different channels obviously influenced the classification selection, we calculated the signal-to-noise ratio (SNR, defined as the ratio of the mean signal to its standard deviation) within each channel (Table 1, **top**, Figure 3). The results showed that the signals derived from channel 3 had a proportionally larger SNR as compared to channel 1 and 2 in both condition MI-simple (channel 1 = 0.99; channel 2 = 0.54; channel 3 = 1.08) and MI-complex (channel 1 = 1.04; channel 2 = 1.23; channel 3 = 1.55).

**Figure 4:**
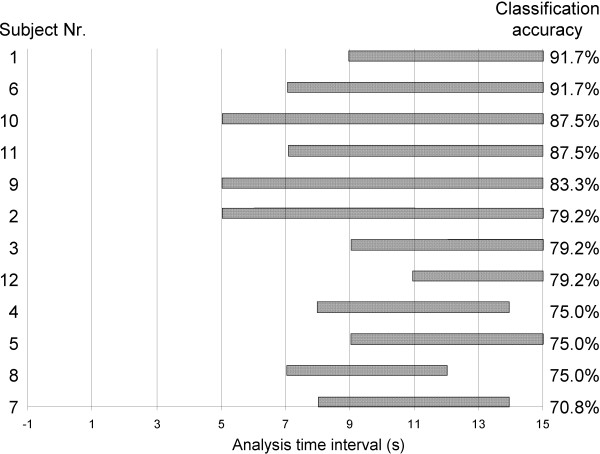
**Analysis time intervals**. Results of the analysis time intervals across subjects ranked by classification accuracy (%). Shown are the ranges of individual analysis intervals used for classification.

**Figure 5:**
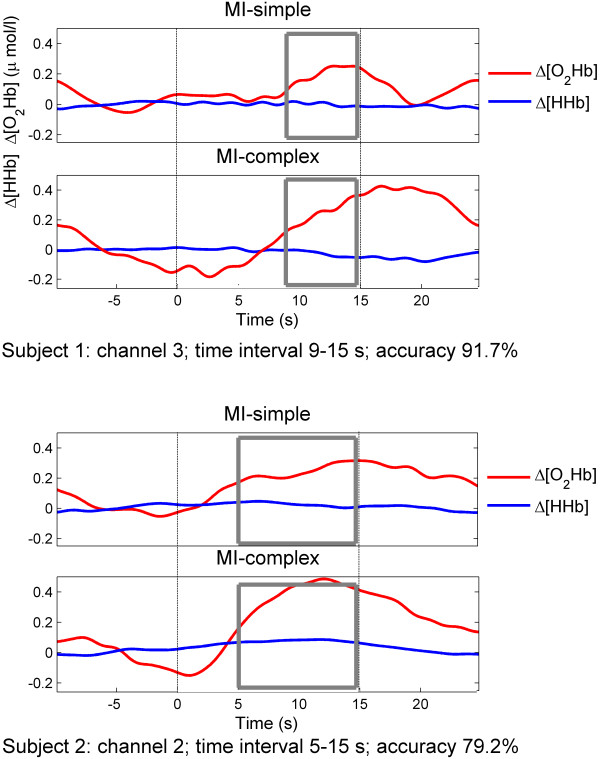
**Sample subjects Δ[O_2_Hb] and Δ[HHb] profile**. Averaged Δ[O_2_Hb] (red) and Δ[HHb] (blue) responses in two sample subjects (subject 1 and 2) corresponding to the classification defined in Table 2. After the rest period (20 s) the on- and offset of the stimulation period (15 s) are indicated by dashed lines from time = 0 - 15 s. The regions highlighted with a box correspond to the time intervals selected for the classification as specified in Table 2.

Further, the response latency in the trial-averaged hemodynamic signals varied among subjects between the 5^th ^to the 15^th ^second of the stimulation phase; accordingly, the best-performing time intervals selected for classification differed between subjects. Figure 4 summarizes the optimal analysis interval lengths across subjects. The figure showed an overall tendency that the longer the time intervals available for classification analysis the higher the classification accuracy ranged. Each horizontal bar represents the analysis interval range for which significant activation was detected for a participant. To illustrate examples of the analysis time intervals within a specific channel, the oxygenation responses of two sample subjects (subject 1 and 2) were plotted (Figure 5); shown are examples of channels 2 and 3 during both conditions MI-simple and MI-complex. The regions highlighted with a box correspond to the time intervals selected for the classification as specified in Table 2. Last, also the four features selected differed between subjects. The most commonly used feature was Δ[O_2_Hb] variance (N = 10 (83%)), followed by mean amplitude (N = 8 (66%)), skewness (N = 6 (12%)) and kurtosis (N = 5 (41%)).

**Table 2:** Classification accuracy for each subject.

Best-performing combination				
**Subject No**.	**Channel**	**Time interval**	**Optimal feature set**	**Classification accuracy**

1	3	9-15 s	Δ[O_2_Hb] mean, variance, skewness, kurtosis	91.7%

2	2	5-15 s	Δ[O_2_Hb] mean, variance	79.2%

3	3	9-15 s	Δ[O_2_Hb] variance, skewness, kurtosis	79.2%

4	2	8-14 s	Δ[O_2_Hb] mean, variance	75.0%

5	3	9-15 s	Δ[O_2_Hb] mean	75.0%

6	3	7-15 s	Δ[O_2_Hb] mean, variance, skewness	91.7%

7	1	8-14 s	Δ[O_2_Hb] skewness	70.8%

8	2	7-12 s	Δ[O_2_Hb] mean, variance	75.0%

9	1	5-15 s	Δ[O_2_Hb] mean, variance	83.3%

10	3	5-15 s	Δ[O_2_Hb]variance, skewness, kurtosis	87.5%

11	3	7-15 s	Δ[O_2_Hb]variance, kurtosis	87.5%

12	2	11-15 s	Δ[O_2_Hb] mean, variance, skewness, kurtosis	79.2%

**Overall**				**81.3 ± 7.0%**

The results are shown for the best-performing combination of one channel, a certain time interval and the optimal feature set for each subject. Classification accuracy was identified over 12 randomised trials by cross validation. Four features were used: mean Δ[O_2_Hb] amplitude, variance, skewness and kurtosis

**Figure 6:**
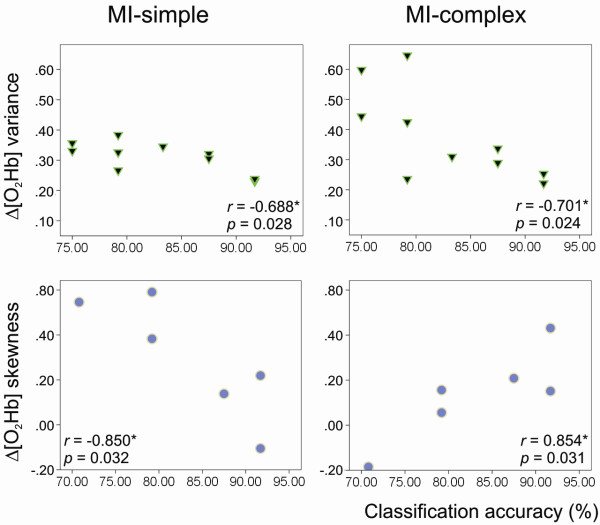
**Correlations between classification accuracy and feature value**. Scatter plots illustrating the correlations between the classification accuracies (%) and the averaged feature values over all trials for each subject (each dot represents one subject, only those subjects are shown for whom the feature was selected for classification). Separate plots are shown for the significant findings in two of the four feature: (Left) Δ[O_2_Hb] variance was negatively correlated with classification accuracy in both conditions (MI-simple: *r *= -0.688*, *p *= 0.028; MI-complex: *r *= -0.701*, *p *= 0.024); (Right) Δ[O_2_Hb] skewness was negatively correlated with classification accuracy in MI-simple (*r *= -0.850*, *p *= 0.032) and positively correlated in MI-complex (*r *= 0.854*, *p *= 0.031).

To determine potential relations between the signal features and the resulting classification accuracy, correlations were calculated between the mean value of the four features and the classification accuracy. As shown in Figure 6, significant correlations were observed in both conditions MI-simple and MI-complex: Δ[O_2_Hb] variance was negatively correlated with classification accuracy in both conditions (MI-simple: *r *= -0.688*, *p *= 0.028; MI-complex: *r *= -0.701*, *p *= 0.024) and Δ[O_2_Hb] skewness was negatively correlated with classification accuracy in MI-simple (*r *= -0.850*, *p *= 0.032) and positively correlated in MI-complex (*r *= 0.854*, *p *= 0.031).

## 5 Discussion

We present results of single-trial classification of MI signals using a novel wireless fNIRS instrument. Our findings show, that using a simple feature combination selected by linear discriminant analysis, it is possible to discriminate between single-trials in response to MI tasks differing in tasks complexity, i.e. simple versus complex tasks. Our results revealed an average accuracy of 81% that was achieved by selecting for each subject a best-performing combination consisting of one channel, a certain time interval and up to four Δ[O_2_Hb] signal features. In the following discussion we address each of these aspects, their limitations for future single-trial classification approaches and their relevance for neurorehabilitation.

### 5.1 Channels selected for classification

As shown in Table 2, the signal locations, i.e. channels selected for optimal classification, differed across subjects. As a result of this subject-to-subject variability, classification in our study required the individual selection of a suitable channel in which an appropriate time interval with significant oxygenation changes was detected in both task conditions MI-simple and MI-complex. This is in line with previous studies which selected channels and/or time intervals for individual subjects [7,8].

In this study, the channel most frequently selected for classification was channel 3 (N = 6 (50%)), followed by channel 2 (N = 4 (33%)) and 1 (N = 2 (16%)). As illustrated in Figure 2, channel 3 was positioned more lateral over the left hemisphere as compared to channel 1 and 2. This might indicate that either the signals obtained from the very lateral positioned part of the sensor, i.e. channel 3, or the cortical areas covered by that part of the sensor were better suitable for discrimination of the presented MI tasks. Using an approximated topographical assumption we suggested that while the medial part of the sensor was detecting signal derived from SMA, the more lateral part was detecting signal located in areas of PMC. Hence, the signals originating from PMC might have been favoured for greater classification accuracy in the given MI tasks in our study. This might have been unexpected considering that channel 3 elicited the smallest oxygenation changes over all subjects both in response to MI-simple and MI-complex (Figure 3). However, the proportionally larger SNR associated with that smaller signal in channel 3 (Table 1) might have allowed for better classification results. Hence, part of the subject-to-subject variability in signal location might be explained by these observations, i.e. indicating that the more lateral the position of a specific sensor channel and the smaller the signal was - accompanied with a good SNR -, the higher the resulting classification accuracy.

Further reasons for this subject-to-subject variability in signal location might be explained by methodological aspects of fNIRS which can be related to sensor positioning. Although, external landmarks can be used for sensor positioning using the international 10-20 system [38,39], these landmarks offer only probabilistic guidelines for individual differences in location. Hence, as with several other non-invasive brain imaging methods (e.g., EEG) anatomical information and variability between individuals are not directly obtained, making the localization of externally recorded signals difficult with respect to the underlying brain. These and the limitation of the usually restricted NIRS sample volume [39] in our study may have lead to differences in exact location of the interrogated tissue from subject to subject. Therefore, by using F3 as landmark, we could only assume to cover secondary motor areas such as SMA or PMC in the individual subjects.

### 5.2 Analysis time intervals selected for classification

Similar to the signal location, the individual time intervals after onset of the stimulation phase that yielded the best classification accuracy differed between subjects from five to eleven seconds (Table 2, Figure 5). Consequently, the analysis time intervals required for the best classification accuracy varied between subjects within a range from four to ten seconds. This time frame is comparable to those reported by Sitaram et al. [4] who required ten seconds of stimulation data in response to MI of finger-tapping and by Tai et al. [7] who choose intervals between four and 19 seconds during positively and negatively-emotional induction tasks. However, it needs to be taken into account that these time intervals were obtained with offline classification, while online classification has been shown to require at least 15 seconds of MI performance [5]. We suggest that the subject-to-subject variations in the selected time intervals are most likely due to individual latency differences in the delay of the Δ[O_2_Hb] response after onset of the imagination task. Part of these subject-to-subject variations might be explained by differences in the cognitive processes underlying MI performance in our experimental tasks. Although, subjects were explicitly instructed to perform kinesthetic MI, i.e. using imagery to imagine how movements feel, instead of visual imagery, i.e. imagine watching oneself performing a task, or any other form of imagination, we can not provide a measure for the individual strategies used. Another explanation might be the training status of our subjects. Although the answers of the VMIQ revealed relatively good imagery ability among subjects, none of them were explicitly trained in the use of MI. Hence, it might be suggested that subject-to-subject variability may have been lower if recorded in experienced or trained subjects.

### 5.3 Δ[O_2_Hb] signal features selected for classification

Previous studies investigating fNIRS single-trial classification reported the use of different signal features and diverse numbers of trials collected per subject. The majority of studies used mean Δ[O_2_Hb] and/or Δ[HHb] amplitude changes in the hemodynamic response and collected from ten trials per subject during MI [5] to 60 trials per subject during emotional induction [7]. The feature set used in our study - Δ[O_2_Hb] mean amplitude, variance, skewness and kurtosis - was chosen from the selection reported by Tai et al. [7] who found classification accuracies between 75% and 94.67% using these features. We hypothesized that using these additional four features, instead of only the mean amplitude, would enhance potential classification accuracies. This was confirmed in some of our subjects which required up to four of the features to reach higher classification accuracies as compared to only using the mean amplitude. Overall, as with channel and time interval selection, subject-to-subject variability was found also in the feature set selection:

• Δ[O_2_Hb] variance (N = 10 (83%)): This feature was selected most frequently indicating that our data contained a large variation in variance between individual signals and between the two task conditions, MI-simple and MI-complex. However, the value of the variance within an individual signal was relatively stable from trial-to-trial, therefore serving a suitable feature for discrimination between the two tasks. Overall subjects, the averaged value of Δ[O_2_Hb] variance revealed a significant negative correlation with the classification accuracies in both conditions, i.e. classification rates improved with decreasing variance (MI-simple: *r *= -0.688*, *p *= 0.028; MI-complex: *r *= -0.701*, *p *= 0.024) (Figure 6). This finding is in line with the tendency that has been observed for the selection of channels (section 5.1), i.e. channels with larger SNR (in particular channel 3) revealed higher classification accuracies.

• Δ[O_2_Hb] mean amplitude (N = 8 (66%)): The mean amplitude as feature reflected those individual time intervals in which both a significant increase within a given condition and a significant difference between the two conditions was found. As shown by the previous studies the mean amplitude is a reliable feature selected for classification, in particular for classification of two different conditions as in our case. In our study, as again discussed for the selection of channels (section 5.1), there was a slight tendency that smaller mean amplitudes did reveal higher classification accuracies, but no significant correlations were found.

• Δ[O_2_Hb] skewness (N = 6 (12%)): Classification rates also improved in relation to skewness. However, the relationship differed between the two conditions. Skewness of signals in response to MI-simple were negatively correlated with increasing accuracy (*r *= -0.850*, *p *= 0.032), i.e. the smaller the value of the skewness the higher the accuracy of classification in a given subject. In contrast, in MI-complex a positive correlation was observed (*r *= 0.854*, *p *= 0.031), i.e. the higher the skewness the higher the accuracy of classification in a given subject (Figure 6). This finding may reflect differences in the shape of the signal between the simple and the complex imagery task. While in response to the simple task, higher accuracies may have favoured a slower signal increase, i.e. the tail on the left side of the probability density function was longer than the right side and the bulk of the values was located to the right of the peak; contrary, in response to the complex task a faster signal increase may have been favoured reflected by a positive skewness, i.e. the tail on the right side was longer than on the left side.

• Δ[O_2_Hb] kurtosis (N = 5 (41%)): The last feature was selected only in a few subjects, but was relevant in these to achieve the reported classification accuracies. No correlations were found with the classification accuracy.

Although the classification accuracies look promising they are nevertheless subject of limitations. We hypothesized that the use of simple feature sets would facilitate potential implementation in future applications. However, due to the observed subject-to-subject variability such an implementation would require quite different feature sets per subject to achieve sufficient classification accuracy. Although, the necessity for individualized classifier training has been recognized as a well-known issue in single-trial classification [4], the following aspects might have accounted for the subject-to-subject variability observed in our study and could be considered in future classification studies:

First, the number of trials on our study was 12 which is comparable to previous studies [7]. However, it is conceivable that the number of features required for individual subjects may have been lower if more trials were collected. On the other side, the experimental length was inherently limited by the repetitive nature of the protocol and the mental demand of the task on the participant. Future study may explore different numbers of trials to find a suitable balance between features needed, classification accuracy and the demand of the task.

Second, subject-to-subject variability in the hemodynamic onset latency in response to MI performance may be improved. The hemodynamic response measured by fNIRS is temporally delayed from the onset of the underlying neural activity about 6 s. Further, it is known that MI signals generally exhibit longer onset latencies as compared to ME signals. Previous studies found that Δ[O_2_Hb] in response to MI increased about 2 s later compared to real movement execution [40]. However, envisioning an application in neural interfaces, MI as mental task therefore still limits the practical use of NIRS based systems. Compared to other mental tasks this delay might be explained by the training status of the individual subject. For example, while mental tasks such as preference decision making [8] or emotional evaluation [7] might be performed more intuitively without training, MI for use in neural interfaces does require considerable training as shown by recent evidence from both neurorehabilitation applications [41] and operating BCIs [42]. It might be therefore suggested that subjects experienced or trained in MI might have elicited faster and less variable responses.

### 5.4 Future work

Considering future applications, while MI training may be possible in most healthy subjects and the majority of patients, some patients, especially those severely impaired, may not provide sufficient cognitive capabilities to train MI. This might further limit the use of MI in neural interfaces as compared to alternative BCI paradigms using more intuitive mental tasks [8]. To evaluate the potential use in a BCI or in neurorehabilitation, it would be therefore necessary to test our classification approach in several patient groups, such as affected by stroke, cerebral palsy, amyotrophic lateral sclerosis, and other motor neuron diseases. Such future work would further require including solutions for the reduction of subject-to-subject variability, such as specifically designed training sessions.

Last, future studies could also address methodological options to reduce the hemodynamic response delay in NIRS signal. A recent example has been given by Cui et al. 2010 [43] who reported that it may be possible to decode the true behavioral state from the measured neural signal - instead of the hemodynamic signal - using fNIRS. The authors reported that using a multivariate pattern classification technique (linear support vector machine, SVM) and systematically evaluation of the performance of different feature spaces (signal history, history gradient, signal and spatial pattern of Δ[O_2_Hb] and Δ[HHb]), the latency to decode a change in behavioral state could be reduced by 50% (from 4.8 s to 2.4 s), which would enhance the feasibility of MI based real-time NIRS applications.

### 5.5 Relevance of MI classification for neurorehabilitation

Our experimental design was motivated by two aspects related to the use of MI as mental task in neurorehabilitation. First, our attempt to classify two tasks differing in complexity was motivated by the known fact that there is a difference in (re)learning a simple as compared to a complex task. One hypothesis is that the cognitive processing demands may be inherently greater for the learning of complex tasks [44]. This has demonstrated the need to use both simple and complex skills in motor-learning research in order to gain further insights into these potentially distinct learning processes and - in our case - the underlying signal features. Therefore, current neurorehabilitation strategies usually address tasks differing in complexity, e.g. fine coordination and precise dexterity versus gross movements, single finger versus whole hand or arm movements or with versus without the use of objects for goal-directed actions such as in our case the keyboard. Thus, we suggested that our approach of evaluation tasks differing in complexity, i.e. both simple and complex finger-tapping tasks for single-trial classification is of relevance for neurorehabilitative applications.

Second, several mental tasks have been recently investigated in the development of neural interfaces, e.g. mental arithmetic tasks [45], language-, visual- and auditory-based imagery tasks or spatial navigation imagery [46]. Those mental tasks are suitable to fulfil the main goals of neural interfaces, i.e. communication such as using spelling devices or the control of external devices such as neuroprostheses. In neurorehabilitation an additional goal is to combine neural interfaces with the training or relearning of impaired motor function [47]. An example for such a combined approach would be a combination of BCI training and physical therapy such as in stroke patients [48]. For such applications, MI has been suggested as a suitable mental task as it - according to the simulation hypothesis - not only activates the impaired motor areas responsible for task execution [11], but also accesses the motor network independently of the impaired function thereby improving recovery [49]. Especially in less severe disabled persons, e.g. in individuals with upper-limb paralysis, MI based BCI systems could be used as tools to recruit and reinforce spared cortical networks by activating the corresponding neural representations. As Dobkin [50] suggested, using such a combined training-BCI approach, researchers and therapists may be able to improve the effects of a rehabilitation treatment aimed at impairment and disability. Further, MI signals may enhance training possibilities by providing insight whether an individual is indeed engaging the network for mental rehearsal. For example, therapists could use the change in the MI signal to get immediate feedback about whether an individual is optimally focussing on the imagined movement thereby monitoring treatment progress. Last, signals derived from MI performance may be used as direct online feedback for the individual. Such feedback may represent the Δ[O_2_Hb] amplitudes of the recruited motor pools elicited in the individual's brain, which in turn may motivate for increased subsequent MI output and improve the timing and completeness of imagined movements. As a result, individuals may regain strength and precision if they can find a way to practise with MI signals thereby accelerating normal recovery.

## 6 Conclusion

To summarize, the results of our single-trial classification showed that using the simple combination set of channels, time intervals and up to four Δ[O_2_Hb] signal features comprising Δ[O_2_Hb] mean signal amplitudes, variance, skewness and kurtosis, it was possible to discriminate single trials of MI tasks differing in complexity, i.e. simple versus complex tasks, over secondary motor areas with an average accuracy of 81%. Although the classification accuracies look promising they are nevertheless subject of subject-to-subject variability and limitations that require further evaluation. Since MI is now applied frequently as a valid tool in neurorehabilitation, the results may be of relevance for future application using MI as mental task in combined approaches of neurorehabilitative training together with BCI use.

## Declaration of competing interests

The authors declare that they have no competing interests.

## Authors' contributions

LH conceived of the study, conducted the fNIRS recordings, carried out the statistical analysis, and drafted the manuscript. MW participated in the design and coordination of the study. Both authors read and approved the final manuscript.

## References

[B1] Wolpaw JR Brain-computer interfaces for communication and control Clinical Neurophysiology 2002 113 6 767 791 10.1016/S1388-2457(02)00057-3 12048038

[B2] Hoshi Y Tamura M Detection of dynamic changes in cerebral oxygenation coupled to neuronal function during mental work in men Neuroscience Letters 1993 150 5 8 10.1016/0304-3940(93)90094-2 8469403

[B3] Muehlemann T Haensse D Wolf M Wireless miniaturized in-vivo near infrared imaging Optics Express 2008 16 14 10323 30 10.1364/OE.16.010323 18607442

[B4] Sitaram R Temporal classification of multichannel near-infrared spectroscopy signals of motor imagery for developing a brain-computer interface NeuroImage 2007 34 4 1416 1427 10.1016/j.neuroimage.2006.11.005 17196832

[B5] Coyle SM Ward TE Markham CM Brain-computer interface using a simplified functional near-infrared spectroscopy system Journal of Neural Engineering 2007 4 3 219 226 10.1088/1741-2560/4/3/007 17873424

[B6] Naito M A Communication Means for Totally Locked-in ALS Patients Based on Changes in Cerebral Blood Volume Measured with Near-Infrared Light IEICE T Inf Syst 2007 90 7 1028 1037

[B7] Tai K Chau T Single-trial classification of NIRS signals during emotional induction tasks: towards a corporeal machine interface Journal of NeuroEngineering and Rehabilitation 2009 6 1 39 10.1186/1743-0003-6-39 19900285 PMC2779792

[B8] Luu S Chau T Decoding subjective preference from single-trial near-infrared spectroscopy signals Journal of Neural Engineering 2009 6 1 016003 10.1088/1741-2560/6/1/016003 19104138

[B9] Jeannerod M Neural Simulation of Action: A Unifying Mechanism for Motor Cognition NeuroImage 2001 14 1 S103 S109 10.1006/nimg.2001.0832 11373140

[B10] Rizzolatti G Fogassi L Gallese V Neurophysiological mechanisms underlying the understanding and imitation of actions Nature Reviews Neuroscience 2001 2 661 670 10.1038/35090060 11533734

[B11] Jeannerod M The representing brain: Neural correlates of motor intention and imagery Behavioural Brain Research 1994 17 187 245 10.1017/S0140525X00034026

[B12] Fadiga L Motor facilitation during action observation: a magnetic stimulation study Journal of Neurophysiology 1995 73 6 2608 2611 7666169 10.1152/jn.1995.73.6.2608

[B13] Decety J Do imagined and executed actions share the same neural substrate? Cognitive Brain Research 1996 3 2 87 93 10.1016/0926-6410(95)00033-X 8713549

[B14] Lotze M Activation of cortical and cerebellar motor areas during executed and imagined hand movements: an fMRI study Journal of Cognitive Neuroscience 1999 11 5 491 501 10.1162/089892999563553 10511638

[B15] Bovend'Eerdt TJ An Integrated Motor Imagery Program to Improve Functional Task Performance in Neurorehabilitation: A Single-Blind Randomized Controlled Trial Archives of Physical Medicine and Rehabilitation 2010 91 6 939 946 10.1016/j.apmr.2010.03.008 20510987

[B16] Pfurtscheller G Brain-computer communication based on the dynamics of brain oscillations Supplements to Clinical Neurophysiology 2004 57 583 91 16106660 10.1016/s1567-424x(09)70398-8

[B17] Andersen R Hwang E Mulliken G Cognitive Neural Prosthetics Annual Review of Psychology 2010 61 1 169 190 10.1146/annurev.psych.093008.100503 19575625 PMC2849803

[B18] Seitz RJ Role of the Premotor Cortex in Recovery From Middle Cerebral Artery Infarction Arch Neurol 1998 55 8 1081 1088 10.1001/archneur.55.8.1081 9708958

[B19] Chollet F The functional anatomy of motor recovery after stroke in humans: a study with positron emission tomography Ann Neurol 1991 29 1 63 71 10.1002/ana.410290112 1996881

[B20] Cao Y Pilot Study of Functional MRI to Assess Cerebral Activation of Motor Function after Poststroke Hemiparesis Stroke 1998 29 1 112 122 10.1161/01.STR.29.1.112 9445338

[B21] Weiller C Individual patterns of functional reorganization in the human cerebral cortex after capsular infarction Ann Neurol 1993 33 2 181 9 10.1002/ana.410330208 8434880

[B22] Holper L Testing the potential of a virtual reality neurorehabilitation system during performance of observation, imagery and imitation of motor actions recorded by wireless functional near-infrared spectroscopy (fNIRS) Journal of NeuroEngineering and Rehabilitation 2010 7 1 57 10.1186/1743-0003-7-57 21122154 PMC3014953

[B23] Holper L Extension of mental preparation positively affects motor imagery: a functional near-infrared spectroscopy study Cortex 2011 in press 10.1016/j.cortex.2011.02.001 21377666

[B24] Oldfield R The assessment and analysis of handedness: the Edinburgh inventory Neuropsychologia 1971 9 1 97 113 10.1016/0028-3932(71)90067-4 5146491

[B25] Isaac A Marks D Russell D An instrument for assessing imagery of movement: The vividness of movement imagery questionnaire (VMIQ) Journal of Mental Imagery 1986 10 4 23 30

[B26] Whetstone T Enhancing Psychomotor Skill Development Through the Use of Mental Practice Journal of Industrial Teacher Education 1995 32 4

[B27] Stinear C Kinesthetic, but not visual, motor imagery modulates corticomotor excitability Experimental Brain Research 2006 168 1 57 164 10.1007/s00221-005-0078-y 16078024

[B28] Neuper C Imagery of motor actions: Differential effects of kinesthetic and visual-motor mode of imagery in single-trial EEG Cognitive Brain Research 2005 25 3 668 677 10.1016/j.cogbrainres.2005.08.014 16236487

[B29] Wolf M Different time evolution of oxyhemoglobin and deoxyhemoglobin concentration changes in the visual and motor cortices during functional stimulation: a near-infrared spectroscopy study NeuroImage 2002 16 3 Pt 1 704 12 12169254 10.1006/nimg.2002.1128

[B30] Jaspers H The ten-twenty electrode system of the International Federation Electroencephalography and Clinical Neurophysiology 1958 10 371 375 10590970

[B31] Chen H Evaluation of the effective connectivity of supplementary motor areas during motor imagery using Granger causality mapping NeuroImage 2009 47 4 1844 1853 10.1016/j.neuroimage.2009.06.026 19540349

[B32] Hanakawa T Functional Properties of Brain Areas Associated With Motor Execution and Imagery Journal of Neurophysiology 2003 89 2 989 1002 12574475 10.1152/jn.00132.2002

[B33] Delpy D Estimation of optical path length through tissue from direct time of flight measurements Physics in Medicine and Biology 1988 33 1433 42 10.1088/0031-9155/33/12/008 3237772

[B34] Wray S Characterization of the near infrared absorption spectra of cytochrome aa3 and haemoglobin for the non-invasive monitoring of cerebral oxygenation Biochimica et Biophysica Acta (BBA) - Bioenergetics 1988 933 1 184 192 10.1016/0005-2728(88)90069-2 2831976

[B35] Zhao H Maps of optical differential pathlength factor of human adult forehead, somatosensory motor and occipital regions at multi-wavelengths in NIR Physics in Medicine and Biology 2002 47 2075 2093 10.1088/0031-9155/47/12/306 12118602

[B36] Liao CH Estimating the Delay of the fMRI Response NeuroImage 2002 16 3 Part 1 593 606 12169246 10.1006/nimg.2002.1096

[B37] Logothetis NK Neurophysiological investigation of the basis of the fMRI signal Nature 2001 412 6843 150 157 10.1038/35084005 11449264

[B38] Homan R Herman J Purdy P Cerebral location of international 10-20 system electrode placement Electroencephalogr Clin Neurophysiol 1987 66 4 376 82 10.1016/0013-4694(87)90206-9 2435517

[B39] Steinmetz H Fürst G Meyer B Craniocerebral topography within the international 10-20 system Electroencephalogr Clin Neurophysiol 1989 72 6 499 506 10.1016/0013-4694(89)90227-7 2471619

[B40] Wriessnegger SC Kurzmann J Neuper C Spatio-temporal differences in brain oxygenation between movement execution and imagery: A multichannel near-infrared spectroscopy study International Journal of Psychophysiology 2008 67 1 54 63 10.1016/j.ijpsycho.2007.10.004 18006099

[B41] Olsson CJ Nyberg L Motor imagery: if you can't do it, you won't think it Scandinavian Journal of Medicine & Science in Sports 2010 20338003 10.1111/j.1600-0838.2010.01101.x

[B42] Grosse-Wentrup M Biased feedback in brain-computer interfaces Journal of NeuroEngineering and Rehabilitation 2010 7 1 34 10.1186/1743-0003-7-34 20659350 PMC2927905

[B43] Cui X Bray S Reiss AL Speeded Near Infrared Spectroscopy (NIRS) Response Detection PLoS ONE 2010 5 11 e15474 10.1371/journal.pone.0015474 21085607 PMC2978722

[B44] Wulf G Shea CH Principles derived from the study of simple skills do not generalize to complex skill learning Psychonomic Bulletin & Review 2002 9 2 185 211 10.3758/BF03196276 12120783

[B45] Bauernfeind G Development, set-up and first results for a one-channel near-infrared spectroscopy system Biomed Tech 2008 53 36 43 10.1515/BMT.2008.005 18251709

[B46] Dyson MF Gan JQ Localisation of cognitive tasks used in EEG-based BCIs Clinical Neurophysiology 2010 121 9 1481 1493 10.1016/j.clinph.2010.03.011 20435514

[B47] Birbaumer N Sauseng P Brain-Computer Interface in Neurorehabilitation Brain-Computer Interfaces 2010 Springer Berlin Heidelberg 155 169

[B48] Broetz D Combination of Brain-Computer Interface Training and Goal-Directed Physical Therapy in Chronic Stroke: A Case Report Neurorehabilitation and Neural Repair 2010 24 7 674 679 10.1177/1545968310368683 20519741

[B49] Sharma N Pomeroy VM Baron J-C Motor Imagery: A Backdoor to the Motor System After Stroke? Stroke 2006 37 7 1941 1952 10.1161/01.STR.0000226902.43357.fc 16741183

[B50] Dobkin BH Brain-computer interface technology as a tool to augment plasticity and outcomes for neurological rehabilitation The Journal of Physiology 2007 579 3 637 642 10.1113/jphysiol.2006.123067 17095557 PMC2151380

